# Skin Wound following Irradiation Aggravates Radiation-Induced Brain Injury in a Mouse Model

**DOI:** 10.3390/ijms241310701

**Published:** 2023-06-27

**Authors:** Mang Xiao, Xianghong Li, Li Wang, Bin Lin, Min Zhai, Lisa Hull, Alex Zizzo, Wanchang Cui, Juliann G. Kiang

**Affiliations:** 1 Scientific Research Department, Armed Forces Radiobiology Research Institute, Uniformed Services University of the Health Sciences, Bethesda, MD 20889, USA; xianghong.li@nih.gov (X.L.); li.wang.ctr1@usuhs.edu (L.W.); bin.lin.ctr@usuhs.edu (B.L.); min.zhai.ctr@usuhs.edu (M.Z.); lisa.hull.ctr@usuhs.edu (L.H.); alex.zizzo@usuhs.edu (A.Z.); wanchang.cui.ctr@usuhs.edu (W.C.); juliann.kiang@usuhs.edu (J.G.K.); 2 Henry M. Jackson Foundation for the Advancement of Military Medicine, Bethesda, MD 20817, USA; 3 Department of Pharmacology and Molecular Therapeutics, Uniformed Services University of the Health Sciences, Bethesda, MD 20814, USA; 4 Department of Medicine, Uniformed Services University of the Health Sciences, Bethesda, MD 20814, USA

**Keywords:** radiation, skin wound, radiation combined injury, inflammation, brain injury, blood–brain barrier leakage, neural cell damage

## Abstract

Radiation injury- and radiation combined with skin injury-induced inflammatory responses in the mouse brain were evaluated in this study. Female B6D2F1/J mice were subjected to a sham, a skin wound (SW), 9.5 Gy ^60^Co total-body gamma irradiation (RI), or 9.5 Gy RI combined with a skin puncture wound (RCI). Survival, body weight, and wound healing were tracked for 30 days, and mouse brain samples were collected on day 30 after SW, RI, RCI, and the sham control. Our results showed that RCI caused more severe animal death and body weight loss compared with RI, and skin wound healing was significantly delayed by RCI compared to SW. RCI and RI increased the chemokines Eotaxin, IP-10, MIG, 6Ckine/Exodus2, MCP-5, and TIMP-1 in the brain compared to SW and the sham control mice, and the Western blot results showed that IP-10 and p21 were significantly upregulated in brain cells post-RI or -RCI. RI and RCI activated both astrocytes and endothelial cells in the mouse brain, subsequently inducing blood–brain barrier (BBB) leakage, as shown by the increased ICAM1 and GFAP proteins in the brain and GFAP in the serum. The Doublecortin (DCX) protein, the “gold standard” for measuring neurogenesis, was significantly downregulated by RI and RCI compared with the sham group. Furthermore, RI and RCI decreased the expression of the neural stem cell marker E-cadherin, the intermediate progenitor marker MASH1, the immature neuron cell marker NeuroD1, and the mature neuron cell marker NeuN, indicating neural cell damage in all development stages after RI and RCI. Immunohistochemistry (IHC) staining further confirmed the significant loss of neural cells in RCI. Our data demonstrated that RI and RCI induced brain injury through inflammatory pathways, and RCI exacerbated neural cell damage more than RI.

## 1. Introduction

The risk of radiation-induced injury has increased due to the expanding of nuclear proliferation, terrorist activities, and the distribution of radioactive materials. Radiation combined injury (RCI), which is radiation exposure coupled with other forms of injury, such as wounds, burns, blasts, trauma, hemorrhage, and/or sepsis, results in more than 60% of injuries after a nuclear explosion [1]. RCI greatly increases the risk of morbidity and mortality when compared to radiation injury (RI) alone [2,3]. The mechanisms by which RI and RCI induce multiple organ injury involving hematopoietic, gastrointestinal and brain injuries are complicated pathophysiological courses [4]. Data from our studies in a mouse model have demonstrated that RCI increased mortality significantly more than RI and delayed wound healing more than in non-irradiated mice with a skin wound (SW) [2]. In addition, radiation countermeasures showing mitigative effects in RI may not have a mitigative function in RCI [2,5], indicating that the mechanisms of RCI may not necessarily and completely be the same as RI, which increases the difficulty in the treatment of RCI. Therefore, RCI has been identified by the National Institute of Allergy and Infectious Diseases (NIAID) as a key topic in need of further study [1,6].

Radiation-induced massive proinflammatory cytokine release (cytokine storm) from different organs and tissues has been reported by many research groups, including ours [3,7,8,9]. Recent studies have reported that radiation exposure can induce brain injuries through the inflammatory signaling pathway [10,11]. A single dose of irradiation from 2 to 10 Gy increases proinflammatory cytokine/chemokine release from injured neural cells [12,13,14], and thereby could mediate inflammatory and immune reactions that subsequently result in brain microglia, oligodendrocyte, endothelial cell and astrocyte activation, as well as apoptosis, necroptosis, and pyroptosis [15]. Astrocytes are the largest population among nonneuronal cells, and represent about 20–50% of brain volume in the central nervous system (CNS), support the neurovascular structure, and provide a cellular link to the neurons [12,16]. Astrocytes and endothelial cells are critical for the generation and maintenance of a functional blood–brain barrier (BBB), the vascular structure that restricts the entry of blood circulation elements into the cerebralis. The BBB and the blood–cerebrospinal fluid barrier (BCSFB) are formed of capillary endothelial cells, which line the walls of blood vessels, surrounded by pericytes and astrocytic perivascular end feet [17]. Once the neurovascular structure is damaged, it can cause BBB and BCSFB leakage and dysfunction that further increase cellular permeation and allow an influx of the unrestricted free penetration of immune cells and leukocytes into the brain parenchyma [18,19]. Consequently, the brain inflammatory response, neural cell damage, and neurogenesis disorders can occur as a result [14,20,21]. The brain is an organ with a very poor regenerative capacity after injury [11].

Radiation-induced brain injury in humans is described in terms of acute injury, early-delayed injury, and late-delayed injury [22]. Acute brain injury manifests in days to weeks, and is generally reversible. In contrast, early-delayed brain injury occurs 1–6 months post-irradiation and late-delayed injury is usually observed >6 months after irradiation [23]; both may cause irreversible injury in the brain. Recently, radiation-induced brain injuries have been reported [11,24,25], and we reported that brain hemorrhage severity was significantly higher in RCI mice than in RI mice due to platelet depletion, decreases in ATP production, AKT activation, and increases in p53 activation and cytokines/chemokines [19]. However, the cellular and molecular mechanisms leading to brain injuries after RCI are poorly understood and have rarely been reported to date. In this study, we evaluated and compared the effects of RI- and RCI-induced neuron inflammation and cytotoxicity on the brain niche, and neuronal cell injury that is considered to be in the early-delayed injury stage, 30 days post-RI and -RCI in a mouse model.

## 2. Results

### 2.1. Survival, Body Weight, and/or Skin Wound Healing after SW, RI, and RCI

**Figure 1:**
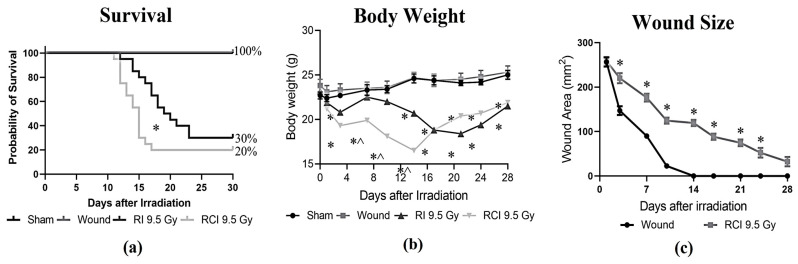
Effects of RI and RCI on 30-day lethality, body weight loss, and wound closure delay. (**a**) Thirty-day survival rate in sham-irradiated, skin wounded, 9.5 Gy RI, and 9.5 Gy RCI. RI and RCI resulted in reduction of survival to 30% and 20%, respectively. SW did not cause animal death. * *p* < 0.05 for RI vs. RCI on day 16 post-irradiation via Log-rank (Mantel–Cox) test. (**b**) RI and RCI resulted in significant body weight loss, and the RCI group lost more body weight than the RI group. * *p* < 0.05 vs. sham and skin wound; ^ *p* < 0.05, RI vs. RCI via two-way ANOVA with Tukey’s multiple comparisons tests. (**c**) RCI delayed wound closure compared with skin wound. * *p* < 0.05 via two-way ANOVA with Tukey’s multiple comparisons tests. N = 20/group in all groups.

Mice receiving 9.5 Gy TBI (RI), RI combined with a skin wound (RCI), and a skin wound without irradiation (SW) were monitored for 30 days, and the percentages of survival on day 30 after insults were calculated. Body weight and skin wound healing were measured on days 0, 3, 7, 10, 14, 21, 24, and 28 post-RI, -RCI and -SW; the data are summarized in Figure 1. In Figure 1a, RI and RCI at 9.5 Gy TBI significantly decreased mouse survival, resulting in 30% survival after RI and 20% survival after RCI, respectively. The 30-day survival rate in the RI and RCI groups showed no significant difference (*p* = 0.76; N = 20). However, RCI caused significantly more animal death than RI on day 16 (*p* = 0.047). SW did not cause any animal death. Body weight was a critical risk indicator of irradiation and was significantly reduced by RI and RCI at all time points, whereas SW did not cause body weight loss compared with the sham-irradiated mice. The body weight in the RCI group of mice further dropped on days 3, 7, 10, and 14 post-TBI and was significantly lower than in the RI group of mice, as shown in Figure 1b (*p* < 0.05). Furthermore, skin wound healing was measured in the SW and RCI groups. Skin wounds were completely closed on day 14 in the SW group; however, RCI significantly delayed wound healing at all time points, and wounds were not closed on day 28, as shown in Figure 1c.

### 2.2. RI- and RCI-Induced Proinflammatory Chemokines Increase in Mouse Brain

**Figure 2:**
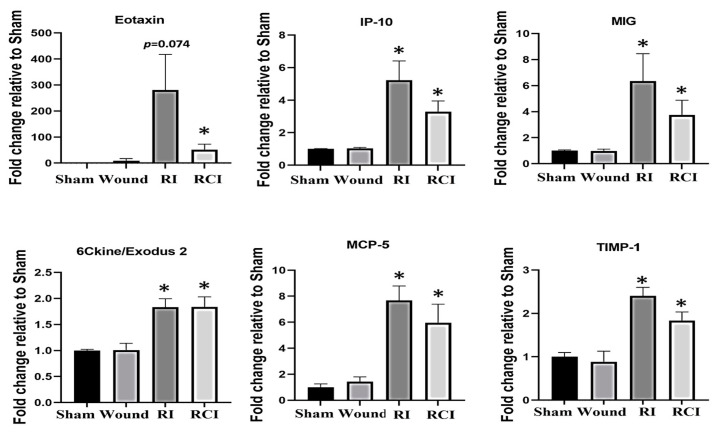
Proinflammatory chemokine increase in mouse brains after skin wound, RI, and RCI. Brain tissues were collected from sham-irradiated, SW, RI, and RCI mice 30 days after TBI. The collected brain tissue lysates (supernatants) were subjected to a cytokine antibody array analysis (with a total of 44 antibodies for cytokines and chemokines) by a company (Eve Technologies, Calgary, AB, Canada). Six chemokines (Eotaxin, IP-10, MIG, 6Ckine, MCP-5, and TIMP-1) were markedly increased in RI and/or RCI groups of mouse brains compared with samples from sham control mouse brains (N = 4–5/group). Data are presented as means ± SEM. * *p* < 0.05 vs. sham-irradiated control group. Wound: skin wound; RI: 9.5 Gy; RCI: 9.5 Gy + SW.

The dysregulation of cytokines and chemokines is a central feature in the development of neuroinflammation. To evaluate the RI- and RCI-induced inflammatory responses, proinflammatory cytokine and chemokine levels in mouse brains were analyzed. Brain tissues were collected from SW, sham-irradiated, RI, or RCI mice 30 days after TBI. The collected brain tissue lysates were subjected to a cytokine antibody array analysis by Eve Technologies (Calgary, AB, Canada) as described in the Materials and Methods section, with each group including 4–5 mice (N = 4–5). Forty-four cytokines/chemokines were analyzed, and the results indicated that six chemokines were increased in samples from the RI and/or RCI groups, whereas no cytokine/chemokine change was observed in the SW mouse brains compared with those samples from the sham control group. The data are summarized in Figure 2. RI and/or RCI significantly increased the chemokines Eotaxin (CCL11), IP-10 (IFN-γ-Inducible Protein 10, CXCL10), MIG (Monokine induced by IFN-γ, CXCL9), 6Ckine/Exodus2 (CCL21), MCP-5 (Monocyte chemotactic protein-5, CCL12), and TIMP-1 (tissue inhibitors of metalloproteinases-1) 1–50-fold in mouse brains compared to the sham control group, respectively. No significant differences in the chemokine levels between the RI and RCI groups, and no cytokine/chemokine level decreases resulting from RI and RCI, were observed.

### 2.3. RI and RCI Upregulate Proinflammatory Factor IP-10 and Cyclin-Dependent Kinase Inhibitor p21 in Mouse Brain

**Figure 3:**
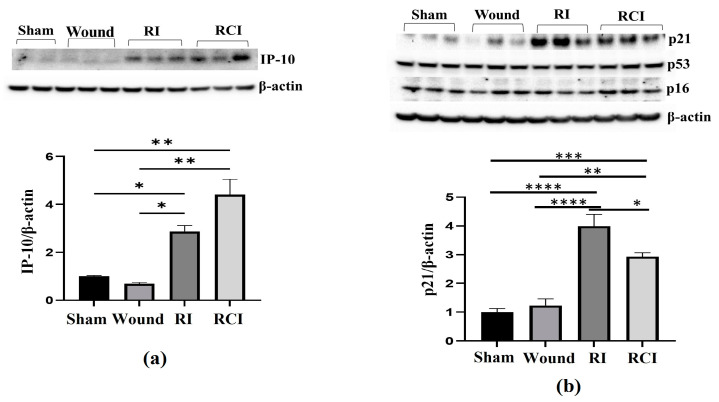
RI and RCI upregulate IP-10 and p21 expression in mouse brain. Mouse brain tissues were collected 30 days after sham-irradiated control, skin wound, RI, and RCI. (**a**) IP-10, (**b**) p16, p21, and p53 were evaluated using an immunoblotting assay. Ratios of IP-10/β-actin and p21/β-actin are shown in histogram panels. Data are presented as means ± SEM. * *p* < 0.05, ** *p* < 0.01, *** *p*< 0.002, **** *p*< 0.001 vs. sham-irradiated control group. Western blot images are presented from one of two independent experiments (N = 4–5/group). Wound: skin wound; RI: 9.5 Gy; RCI: 9.5 Gy + SW.

To further confirm the RI- and RCI-induced inflammation and toxicity in mouse brain cells, we examined the IP-10 expression in the mouse brain samples since this chemokine is expressed by neural cells, glia, and stromal cells and plays a critical role in controlling the entry of leukocyte subsets into the brain after injury [26]. An immunoblotting assay was conducted in samples from 30-day surviving animals, and the results are shown in Figure 3a. The Western blot image shows that the IP-10 protein level was significantly increased by RI and RCI compared with the sham and SW group of samples; the data are further summarized in a histogram and indicate the highest level of IP-10 in the RCI group. Next, the major senescence regulator [27,28] p16INK4A (p16), p53, phosphorylated p53 (ser 15), and p21 (CDKN1A) were evaluated via immunoblotting and the resulting data are shown in Figure 3b. A significant increase in p21 was observed in both the RI and RCI groups, but the protein levels of p16 and p53 were not changed and phosphorylated p53 (ser 15) was not observed in all the group samples. 

### 2.4. RI and RCI Induce Mouse Blood–Brain Barrier (BBB) Leakage

**Figure 4:**
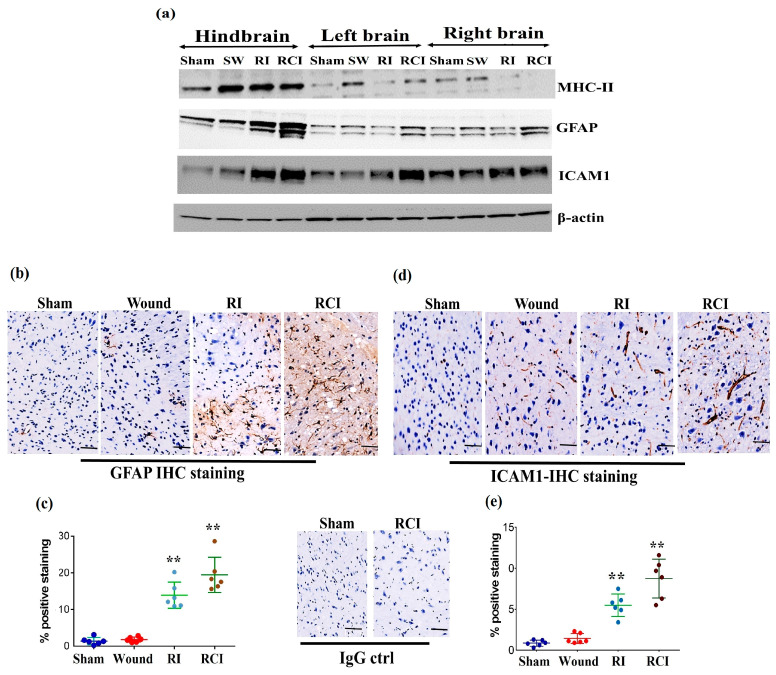
RI and RCI induce microglial, endothelial cell, and astrocyte activation. (**a**) Mouse brain tissues were collected from left brain, right brain and hindbrain 30 days after TBI. The microglial activation marker MHC-II, the astrocyte activation marker GFAP, and the neuro-endothelial cell activation marker ICAM1 were examined via immunoblotting. Data are presented from one of two independent experiments. (**b**–**e**) Immunohistochemistry (IHC) staining of brains with anti-GFAP and anti-ICAM1 antibodies. Mouse brain specimens from sham-irradiated control, SW, RI, and RCI were stained with (**b**) anti-GFAP or (**d**) anti-ICAM1; scale bar = 25 µm. Brown color indicates positive staining and IgG control antibodies show negative staining. Quantification of GFAP IHC staining is shown in (**c**) and ICAM1 IHC staining is shown in (**e**). Three animals/group and two slides/animal. Data are presented as means ± SD. ** *p* < 0.01 vs. sham control group. Note that RCI group has the highest % of GFAP- and ICAM1-positive cells, with an enlarged cell size. SW or Wound: skin wound; RI: 9.5 Gy; RCI: 9.5 Gy + SW.

Radiation-induced inflammatory response in the mouse brain could cause microglial, endothelial cell, and astrocyte activation and subsequently result in neurovascular structure damage, as well as BBB and BCSFB leakage and dysfunction. To evaluate the function of the BBB in the mouse brain after a skin wound, RI, and RCI, the microglial activation marker MHC (Major histocompatibility complex)-II, the astrocyte activation marker GFAP (Glial fibrillary acidic protein), and the neuroendothelial cell activation marker ICAM1 (Intercellular adhesion molecule-1) were examined in mouse brain samples. The representative immunoblotting results are shown in Figure 4a. In comparison with the right- and left-brain samples, the hindbrain samples highly expressed MHC-II after SW, RI, and RCI, whereas only the SW group showed increased MHC-II levels in right and left brain. ICAM1 was upregulated in all brain regions after RI and RCI; GFAP expression increased in the hindbrain samples after RI and RCI, as well as in the right- and left-brain samples after RCI. The hindbrain samples showed the highest expression levels of these three proteins compared to the samples from the right and left brains after wound alone, RI, and/or RCI compared with brain samples from the sham-irradiated control.

The expressions of GFAP and ICAM1 in hindbrain tissue were also confirmed via immunohistochemistry (IHC) staining as described in the Materials and Methods section. Sample slides from three mice/group were stained with specific antibodies, and two slides from each mouse were counted. The images in Figure 4b show the GFAP expression in representative mouse brain tissue slides from different groups using IHC staining. The GFAP-positive stained cells (in brown color) were significantly increased with enlarged cell sizes in the RI and RCI groups compared with the sham-irradiated and wound-alone groups. The data are summarized in a histogram (Figure 4c). GFAP-positive staining was significantly increased by RI (13.0 ± 3.0%) and RCI (19.0 ± 3.8%) compared to the sham control and wound-alone groups (1.0 ± 0.7% and 2.0 ± 0.5% of GFAP-positive staining, respectively; *p* < 0.01). The RCI group had the highest proportion of GFAP-positive staining compared with the other groups. ICAM1 expression in mouse brain tissues from the different groups was also examined using IHC staining, and images of the slides from a representative mouse brain tissue are shown in Figure 4d. ICAM1 expression was evaluated and ICAM1-positive staining cells (in brown color) were significantly increased, with remarkably enlarged cell sizes, in the RI and RCI groups compared with sham-irradiated and wound-alone groups. The data are summarized in a histogram (Figure 4e). ICAM1-positive staining was significantly higher in the RI group (6.0 ± 2.2%) and the RCI group (9.0 ± 3.5%) compared with the sham control and wound-alone groups (1.0 ± 0.5% and 2.0 ± 1.0% ICAM1-positive staining, respectively; *p* < 0.01). The RCI group had the highest proportion of ICAM1-positive staining, with bigger cell sizes than the other groups.

### 2.5. RCI Induced BBB Leakage, as Shown by GFAP Increase in Mouse Serum

**Figure 5:**
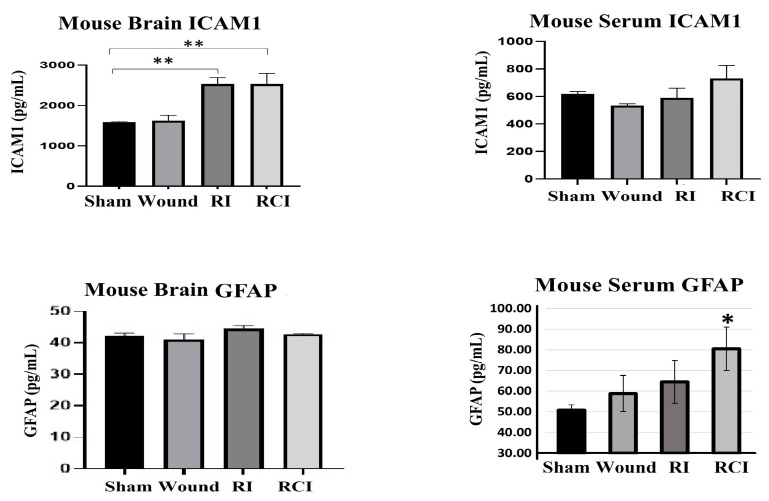
RI and RCI induce mouse blood–brain barrier (BBB) leakage. Levels of ICAM1 and GFAP in mouse brain tissue and serum from sham-irradiated control, skin wound, RI, and RCI mice were measured via ELISA (N = 4–5/group). Data are presented as means ± SD. * *p*< 0.05; ** *p* < 0.01 vs. sham-irradiated control group. Wound: skin wound; RI: 9.5 Gy; RCI: 9.5 Gy + SW.

Furthermore, the levels of ICAM1 and GFAP in the mouse brain and serum from the sham-irradiated control, wound alone, RI, and RCI mice were measured via ELISA (Figure 5; N = 4–5 per group). Serum GFAP is a well-known clinical biomarker of BBB dysfunction, since the GFAP level in healthy mouse serum is relatively low and it will leak from the brain into the serum after BBB damage. The resulting data are shown in Figure 5. RI and RCI significantly increased ICAM1 in the mouse brains; no GFAP level change was observed in the brain cells from different groups. However, the levels of GFAP in the serum of both the wound-alone and RI groups showed varying degrees of increase, with significant elevation observed in the RCI group compared to the serum from sham-irradiated control mice. In contrast, significant changes in serum ICAM1 levels were not observed in the different groups.

### 2.6. RCI Causes More Severe Neural Cell Loss Than RI

**Figure 6:**
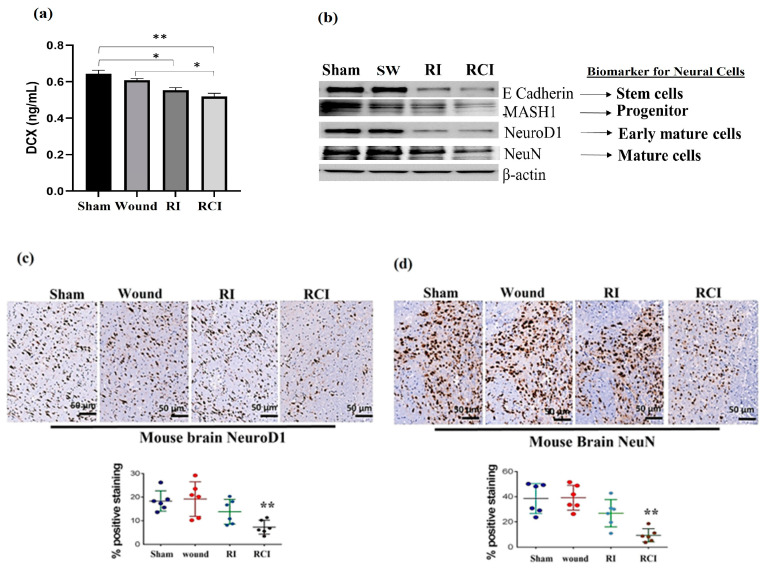
RI and RCI inhibit neurogenesis and decrease neuron cell numbers. (**a**) Levels of DCX in mouse brain from sham-irradiated control, SW, RI, and RCI mice were measured via ELISA (N = 4–5/group). Data are presented as means ± SD. * *p*< 0.05; ** *p* < 0.01 vs. sham-irradiated control group. Wound: skin wound; RI: 9.5 Gy; RCI: 9.5 Gy + SW. (**b**) Neuron cell markers at different development stages were measured via immunoblotting assay with specific antibodies, including anti-E Cadherin (very early stage cell marker), anti-MASH1 (progenitor cell maker), anti- NeuroD1 (early mature neural cell maker), and anti-NeuN (mature neural cell maker). Data are presented from one of two independent experiments. Mouse brain specimens from sham-irradiated control, SW, RI, and RCI. Immunohistochemistry (IHC) staining of brains with anti-NeuroD1 (**c**) or anti- NeuN (**d**) antibodies was performed. Brown color indicates positive staining. Scale bar = 50 µm. Quantification of NeuroD1 and NeuN IHC staining ia shown in the histogram panels, respectively (3 animals/group and 2 slides/animal). Data are presented as means ± SD. **, *p* < 0.01 vs. sham-irradiated control group. Note that RCI group has significantly decreased % of neuron cells. SW or Wound: skin wound; RI: 9.5 Gy; RCI: 9.5 Gy + SW.

Doublecortin (DCX) is a determinant factor in brain growth that is expressed in various regions of the developing nervous system. To evaluate the toxicity of RI and RCI in mouse neuron cells, we determined the DCX levels in mouse brains using ELISA (N = 4–5 per group), and Figure 6a shows the resulting data. RI and RCI significantly decreased the DCX protein level, and RCI caused the greatest reduction in DCX in the mouse brain samples.

Next, the different stages of neural cell population were evaluated via immunoblotting assay and IHC staining using specific antibodies, including anti-E Cadherin (epithelial cadherin, a very early neural cell marker), anti-MASH1 (a progenitor cell maker), anti-NeuroD1 (an early mature neural cell maker) and anti-NeuN (a mature neural cell maker), as described in the Materials and Methods section. RI and RCI decreased neural cell levels at all stages, as shown in the Western blot data (Figure 6b). These results were further confirmed via IHC staining, and the results are shown in Figure 6c,d. The slides from the mouse hindbrain tissues were stained using anti-NeuroD1 or anti-NeuN antibodies, with three mice/group and two slides per mouse examined, and the positive staining was shown as a brown color. The percentages of early mature (Figure 6c, NeuroD1-positive) and mature (Figure 6d, NeuN-positive) neural cells were significantly decreased by RCI in the mouse brain samples, as shown in the representative images, and the data are summarized in histograms (N = three mice/group and two slides/mouse). RCI decreased NeuroD1-positive staining from 19.0 ± 5.6% (sham control group) to 5.0 ± 4.3% (RCI group), and NeuN-positive cells from 39.7 ± 9.0% (sham control group) to 10.0 ± 6.7% (RCI group), respectively.

## 3. Discussion

Our previous research demonstrated that radiation combined injury (RCI) leads to higher mouse mortality rates, delayed wound healing, and organ dysfunction compared to radiation injury (RI) or physical trauma alone [2]. In addition, the differences in the pathologic responses to hematopoietic and gastrointestinal injuries induced by RI and RCI, as well as the mitigative efficacy of radiation countermeasures in RI and RCI, were also observed [2,29]. These findings suggest that the mechanisms underlying RI and RCI may not be entirely the same. Recent studies have suggested that inflammatory signaling pathways play a crucial role in the pathogenesis of radiation-induced brain injuries. [10,21,25]. However, the cellular and molecular mechanisms by which RCI-induced brain injuries occur are still poorly understood and have rarely been reported to date. In this study, we evaluated and compared the cytotoxic effects of RI and RCI on mouse neuronal cell death and brain injury, including BBB dysfunction in the early-delayed term [22], namely 30 days post-RI or -RCI.

We measured the levels of 44 cytokines/chemokines in the brains of mice subjected to RI, RCI, skin wound (SW), or sham-irradiation, and compared the levels between the groups using statistical analysis. Our results showed that, 30 days after RI and RCI, 6 out of 44 cytokines/chemokines levels were significantly elevated in the mouse brains compared with samples from sham-irradiated control. All six elevated proinflammatory factors were chemokines, including Eotaxin, IP-10, MIG, MCP-5, and TIMP-1, as shown in Figure 2. The expression of cytokines/chemokines is almost absent in the resting CNS, but can be highly upregulated during inflammation [30]. Various types of inflammatory chemokine, including CC/CXC motifs, are produced by different CNS cells, such as microglia, astrocytes, neuronal cells, and endothelial cells, and as key signaling molecules in neuroinflammatory processes [31]. We further examined IP-10 expression in mouse brain cells using an immunoblotting assay and observed significant upregulation of IP-10 after RI and RCI. IP-10 belongs to the CXC chemokine family and is expressed by neurons, glia, and stromal cells in response to interferon-γ (IFN-γ) [32,33]. Increased IP-10 levels suggest that inflammatory cells may be migrating into the CNS [34]. In addition, IFN-γ-induced MIG (CXCL9) [35] was also increased by RI and RCI. Our recent study reported radiation-induced IFN-γ upregulation in mouse bone marrow through IL-18 activity [36]. Consistent with the current results in the brain, this indicates that IFN-γ may play an important role in radiation-induced multiple-organ injury. Eotaxin produced by activated mast cells contributes to inflammatory responses in brain disorders [37]. In this study, RCI, but not RI, increased Eotaxin 50-fold in brain tissues compared to the sham control. 6Ckine/Exodus 2 is an unusual chemokine that attracts T cells [38], and the inflammatory chemokine MCP-5 (CcL12) can be stimulated by hypoxia and ischemia in human and mouse brain astrocytes [39]. A recent study suggested that TIMP-1 is an early biomarker for the presence and extent of brain injury in newborns [40]. Thus, the increased expression of these chemokines indicates the presence of severe neuroinflammation and cell damage in these mouse brains 30 days after RI and RCI. The reason that the chemokine but not cytokine expression increased in mouse brains 30 days after RI and RCI is not clear yet, but it may be associated with different stages of brain injury since multiple cytokines, including the increased interleukins, have been reported by other groups at early time points [19,21]. While cytokines and chemokines are primarily involved in mediating inflammation, chemokines and their receptors are also essential in facilitating communication between neurons and inflammatory cells, and they play a crucial role in directing immune cells to the brain [41]. We need to be mindful that the specific chemokines associated with brain impairment caused by either RI or RCI are still unclear, and further studies are required to unravel this information.

To further understand the inflammatory signaling in RI- and RCI-induced mouse brain injury, we examined the major senescence/apoptosis regulator p16, p53, phosphorylated p53 (ser 15), and p21 in mouse brains. Our data demonstrated that p21 was significantly upregulated in mouse brains 30 days after RI and RCI, indicating a potential role of p21 in RI- and RCI-induced brain injury. Since p21 has multiple functions that largely depend on direct p21 signal network interactions and the time [42], further investigation regarding RI- and RCI-induced p21 expression in mouse brains is needed.

Our results also showed that RI and RCI were highly upregulated the astrocytes activation marker GFAP and the endothelial cell activation marker ICAM1 in all brain tissues from the left- and right-side brain and the hindbrain (Figure 4). GFAP is a monomeric intermediate filament protein found in the astroglia cytoskeleton with a very low level in healthy animal brains, and is rarely found outside the CNS [43]; these characteristics suggest that an increased GFAP level in the brain and blood might be a biomarker for brain injury and BBB leakage, as it is known that the GFAP protein can be released into the blood after brain cell damage, leading to increased BBB permeability [44]. To confirm BBB dysfunction in these injured mouse brains, we evaluated the GFAP and ICAM1 levels in mouse brains and serum 30 days after SW, RI, and RCI using ELISA. The data in Figure 5 show that the serum GFAP was elevated by all injuries to a different degree, and RCI significantly increased the GFAP level compared to the sham-irradiated mouse serum samples, suggesting that RCI has caused severe BBB leakage in the mouse brain, and such leakage remains up to day 30 after insults. ICAM1 is a cell surface glycoprotein and member of the adhesion immunoglobulin superfamily; it is usually expressed at a low basal level in healthy animals, but is upregulated by inflamed or damaged vascular endothelium. ICAM1 plays a key role in regulating leukocyte and immune cell recruitment from the circulation to sites of inflammation, and guides leukocytes crossing the endothelial layer via BBB breakdown [45,46,47]. ICAM1 was highly expressed in mouse brain endothelial cells with enlarged cell size in the RI and RCI groups, as shown in the IHC-stained brain slides (Figure 4d,e and Figure 5). Again, the RCI group had the highest percentage of ICAM1-positive staining. Together, the increased GFAP and ICAM1 expression in mouse brains and GFAP in the serum suggests the presence of RI- and RCI-induced astrocyte and endothelial cell activation, and BBB dysfunction. RCI caused the most severe cerebral vascular injury and BBB leakage in this mouse model. The results suggest that astrocytes and endothelial cells play a significant role in regulating the integrity and proper biological function of neurons, although it is important to note that not all aspects of this regulation have been fully elucidated.

Finally, RI and RCI inhibited neurogenesis, as shown by the reduction in DCX protein and damaged neural cells in all developing stages. DCX is a determinant factor in the growing brain and is expressed in various regions of the developing nervous system with cell proliferation during neurogenesis and neuronal migration [48]. Our data demonstrated a significant decrease in DCX in mouse brain cells 30 days after RI and RCI, indicating that neuron cell proliferation and generation were affected in these mice. The brain is an organ with a very poor regenerative capacity after injury. RI and RCI further inhibited neurogenesis, and the percentages of early mature (NeuroD1-positive) and mature (NeuN-positive) neural cells were significantly decreased in RCI mouse brain samples, as shown in IHC stained images (Figure 6). Thus, the data from the current study, for the first time, demonstrate disparity in the responses to RI- and RCI-induced inflammatory signaling in mouse brains, with more severe brain injury in RCI mice, suggesting that the mechanisms underlying RI and RCI are not completely the same. It appears that DCX is involved in regulating neuronal cell proliferation and contributes to the differential response observed between RI and RCI. It can be speculated that AKT/MAPK cross-talk and p53, along with other not-yet-identified elements, could be significant factors leading to this observed disparity [19]. Since RCI has a high incidence rate after nuclear explosion and there are no FDA-approved countermeasures available for RCI to date, the results from our study may provide insight into RCI-caused toxicity in brain injury and suggest potential therapeutic targets for the treatment of RCI.

## 4. Materials and Methods

### 4.1. Ethics Statement

Animals were housed in an Association for Assessment and Accreditation of Laboratory Animal Care (AAALAC)-approved facility at the Uniformed Services University of the Health Sciences (USUHS), Bethesda, MD, USA, and all animal-handling procedures were performed in compliance with guidelines of the National Research Council (2011). All animal experiments were reviewed and approved by the USUHS Institutional Animal Care and Use Committee (IACUC protocol # AFR-20-021). All procedures and animal handling were carried out in accordance with the USUHS Department for Laboratory Animal Resources (DLAR) guidelines.

### 4.2. Mice and Animal Care

B6D2F1/J female mice (12–13 weeks old) were received from Jackson Laboratory (Bar Harbor, ME, USA) [1]. Upon arrival, all of the mice were allowed to acclimate to their new surroundings for 72 h. They were then randomized for each experimental group with 5 mice per cage. The animal room was maintained at 23 °C ± 3 °C with 50% ± 20% relative humidity on a 12:12 h light–dark schedule. Commercial rodent feed (Envigo Teklad Rodent Diet; Envigo Inc. Indianapolis, IN, USA) and acidified water (pH 2.5–3.0) used to control opportunistic infections were available ad libitum to all animals.

### 4.3. Total-Body Irradiation

The B6D2F1/J female mice received total-body irradiation (TBI) from a bilateral radiation field at the Armed Forces Radiobiology Research Institute (AFRRI)’s ^60^Co facility [1]. An alanine/electron spin resonance (ESR) dosimetry system (American Society for Testing and Materials, Standard E 1607; ASTI International, Philadelphia, PA, USA) was used to measure dose rates (to water) in the cores of acrylic mouse phantoms. Prior to TBI, all mice were awake and placed in ventilated plexiglass containers (four mice per box with separate compartments for each animal), and a single, mid-line tissue dose of 9.5 Gy was delivered at an approximate dose rate of 0.4 Gy/min. Animals in the control and wound-alone groups were sham-irradiated and treated in the same manner as the irradiated animals, except with no exposure to the ^60^Co source [49]. They remained in the cobalt staging room during irradiation. The day of irradiation was considered day 0.

### 4.4. Skin Wounding

The skin wounding procedure was performed as described previously [2,3] and comprised two steps (depilation and skin wounding), both of which were performed under anesthesia via isoflurane inhalation. In preparation for skin wounding, the dorsal fur was shaved using an electric hair clipper two days before TBI. A 250–300-mm^2^ circular wound was created using a 70% ethanol-sterilized steel punch through the anterior-dorsal skin fold and underlying panniculus carnosus muscle (between the shoulder blades) within 1–2 h after sham irradiation or TBI for animals in wound-alone and RCI groups, respectively. Subsequent to the skin punch, these non-lethal wounds were left open to the environment, and all animals were placed in autoclaved clean cages containing autoclaved bedding. All mice subjected to skin injury were also given 0.5 mL of acetaminophen solution (150 mg/kg in saline, OFIRMEVt injection, NDC 43825-102-01; Mallinckrodte Pharmaceuticals, Hazelwood, MO, USA) intraperitoneally (I.P.) immediately after skin injury to alleviate pain. Acetaminophen was used to alleviate the acute pain induced by skin wounding and minimize distress in skin wounded-alone (SW) and RCI mice.

### 4.5. Survival, Body Weight, and Wound Healing

Mice receiving 9.5 Gy TBI (RI), RI combined with skin wound (RCI), and skin wound without irradiation (SW) were closely monitored for 30 days as indicated in a previous report [2]. Morbid animals were examined at least three times daily, and the moribund animals considered to have met the endpoint criteria were euthanized via CO_2_ inhalation plus confirmatory cervical dislocation. The percentage of surviving mice was recorded, and a Kaplan–Meier survival curve was plotted (N = 20/group). Body weight and wound size measurements were also performed on each animal for 30 days. The basal body weight and skin wound size were measured immediately following irradiation (day 0), and on days 1, 3, 7, 10, 14, 21, 24, and 28 post-insult. Each wound area was calculated according to previous reporting [2, 3]: wound area = π × A/2 × B/2 (A and B represent diameters at right angles to each other).

### 4.6. Brain Tissue Collection

Mouse brain samples were collected from euthanized animals 30 days after sham-irradiation, SW, RI, or RCI (N = 4–6/group). Briefly, the whole brain was first separated into forebrain (right and left side) and hindbrain pieces with a razor blade, and then, stored at −80 °C until further use.

### 4.7. Brain Cytokine Antibody Array

The frozen mouse brain tissues were homogenized in RIPA lysis and extract buffer (Thermo Scientific, Rockford, IL, USA) supplemented with a HaltTM protease inhibitor Cocktail (Thermo Scientific, Rockford, IL, USA) using the Stomachert 80 Biomaster Lab System (Seward Laboratory Systems, Port St. Lucie, FL, USA) following the manufacturer’s recommendations. After 15 min centrifugation at 12,000× *g*, the supernatant was collected and protein concentrations were determined using a BCA assay kit (Thermo Scientific, Rockford, IL, USA). The collected supernatants (brain tissue lysates) were subjected to a cytokine antibody array analysis by Eve Technologies Company (Calgary, AB, Canada). In brief, 100 µL of brain tissue lysate at a concentration of 4 µg/µL was subjected to mouse cytokine/chemokine 44-plex discovery assay^®^ array (MD44). A total of 44 cytokines/chemokines, including Eotaxin, Erythropoietin, 6Ckine, Fractalkine, G-CSF, GM-CSF, IFNB1, IFNγ, IL-1α, IL-1β, IL-2, IL-3, IL-4, IL-5, IL-6, IL-7, IL-9, IL-10, IL-11, IL-12p40, IL-12p70, IL-13, IL-15, IL-16, IL-17, IL-20, IP-10, KC, LIF, LIX, MCP-1, MCP-5, M-CSF, MDC, MIG, MIP-1α, MIP-1β, MIP-2, MIP-3α, MIP-3B, RANTES, TARC, TIMP-1, TNFα, and VEGF-A, were measured.

### 4.8. Protein Extraction and Immunoblotting

The frozen mouse brain tissues were homogenized in N-PER Neuronal Protein Extraction Reagent (Thermo Scientific, Rockford, IL, USA) supplemented with a protease inhibitor tablet using a tissue homogenizer (FastPrep-24e; MP Biomedicals, Solon, OH, USA), following manufacturer’s recommendations. After 15 min centrifugation at 12,000× *g*, the supernatant was collected and protein concentrations were determined using a BCA assay kit (Thermo Scientific, Rockford, IL, USA). The collected homogenates were denatured in Laemmli buffer and loaded for SDS-PAGE electrophoresis. Following the standard procedures, immunoblotting was then performed using an enhanced chemiluminescence kit (Thermo Fisher Scientifice Inc., Rockford, IL, USA). The images were captured using a ChemiDoc MP Imaging System (Biorad, Hercules, CA, USA). Antibodies for GFAP (ab7260, 1:5000 dilution), ICAM 1 (ab179707, 1:1000 dilution), IP10 (ab9938, 0.15 µg/mL), p21 (ab188224, 1:1000 dilution), p53 (ab131442, 1:500 dilution), MASH 1 (ab211327, 1:1000 dilution), E-cadherin (ab212059, 1:1000 dilution), NeuroD 1 (ab213725, 1:1000 dilution), and NeuN (ab177487, 1:2000 dilution) were purchased from Abcam (Cambridge, MA, USA), MHC-II (sc59322, 1:200 dilution) from Santa Cruz Biotechnology, p16 (PA5-20379, 2 µg/mL) and from Invitrogen (Carlsbad, CA, USA). β-actin (A5441, 1:10,000 dilution) was obtained from Sigma-Aldrich (St Louis, MO, USA).

Western blots were stripped using a Restore™ PLUS Western Blot Stripping Buffer (Thermo Fisher Scientific Inc., Rockford, IL, USA) for 30 min at room temperature, followed by thorough washing with TBST (3× for 15 min each) prior to re-probing the blot with different antibodies.

### 4.9. Brain Immunohistochemistry (IHC) Staining

The mouse brain samples were fixed in 10% formalin, embedded in paraffin, and cut into 5 µm sections per slide. IHC staining was conducted via antigen retrieval in a citrate buffer (pH 6.0) at 100 °C for 20 min, followed by gradual cooling to room temperature. After quenching the endogenous peroxidase activity using BloxAll solution (Vector Laboratories, Inc., Burlingame, CA, USA), the specimens were blocked with 2.5% normal serum (Vector Laboratories, Inc.), and then, incubated at 4 °C overnight with primary antibodies (anti-GFAP antibody, abcam ab7260, 1:2000 dilution; anti-ICAM1 antibody, abcam ab179707, 1:2000 dilution; anti-NeuN antibody, abcam ab177487, 1:3000 dilution; anti-NeuroD1 antibody, abcam ab213725B6688, 1:1000 dilution; and rabbit IgG isotype control antibody, abcam ab37415). The specimens were washed and subsequently incubated using ImmPRESS HRP IgG Polymer Detection Kits (Vector Laboratories, Inc.), and the ImmPACT DAB Kit (Vector Laboratories, Inc.) was used to develop the substrate to the desired stain intensity. The slides were counterstained with hematoxylin, and images were scanned using a Zeiss Axio Scan.Z1 system.

The IHC-positive staining was quantified using ImageJ Fiji software according to our published protocol [27]. All the slides prepared from 3 mice/group with 2 slides/mouse were examined. Two random areas were chosen from each brain sample slide. The DAB color was deconvoluted, and its threshold value was adjusted to achieve the optimal representation of positive staining. Using the same settings, the percentage of positive staining area in each image was quantified.

### 4.10. Enzyme-Linked Immune Sorbent Assay (ELISA)

GFAP and ICAM1 in mouse brain and serum, and DCX in the brain were quantitated via ELISA. The mouse ELISA kit for GFAP (CSB-E08603m) was purchased from CUSABIO (Houston, TX, USA), ICAM1 (ab100688) was purchased from abcam, and DCX (MBS451857) was purchased from MyBioSource. The GFAP, ICAM1, and DXC levels in the serum and/or brain tissue were determined in duplicate following assay instructions provided by the manufacturers (N = 5 mice/group). Briefly, 50 µL/well of mouse serum (1:25 dilution for GFAP; 1:50 dilution for ICAM1) or 50 µg protein (for ICAM1), 5µg protein (for GFAP), and 75 µg protein (for DXC) from each brain sample were added to a 96-microwell plate precoated with anti-mouse GFAP, ICAM1, or DXC antibody, respectively. The plates were incubated for 2 h. After removing the liquid, the biotin antibody was added to each well and incubated for 1 h. After washing, the HRP avidin was added to the microwells and incubated for 1 h. After another washing stage, the substrate reagent was added to each well and incubated for 30 min. An acid solution was added to each microwell to terminate the enzyme reaction and stabilize the color development. The optical density (O.D.) of each microwell was measured at 450 nm using a microplate reader. The minimum detection limit for mouse GFAP was 3.12 pg/mL, for ICAM1 was 24.69 pg/mL, and for DXC was 0.156 ng/mL.

### 4.11. Statistical Analysis

The 30-day survival of mice was analyzed using Kaplan–Meier analysis. Fisher’s exact test was used to compare survival among groups at the end of 30 days after TBI, and *p* < 0.05 was taken as statistically significant. For other cell biology data, differences between means were compared via analysis of variance (ANOVA) and Student’s *t* tests. *p* < 0.05 was considered statistically significant. Results are presented as means ± standard deviation (SD) or standard error of the mean (SEM).

## 5. Conclusions

Previous studies have suggested that radiation-induced brain injury only occurs at very high doses of radiation exposure [23]. However, our current study demonstrated that 9.5 Gy RI- and RCI-induced inflammation still resulted in brain injury in mice. The neuroinflammation and brain injury observed in our study were associated with elevated chemokine expression in mouse brains up to 30 days after both RI and RCI. The mechanisms underlying RI- and RCI-induced brain injury involved multiple cell types, including astrocytes, microglia, endothelial cells, and neurons, which initiated and responded to inflammatory cascades, ultimately leading to progressive neurological damage. Notably, our study found that RCI caused more severe and long-lasting neuroinflammation and BBB leakage than RI, resulting in more pronounced inhibition of neurogenesis and greater neural cell damage, which might contribute to higher animal mortality after RCI compared to RI. These findings suggest the notion that the mechanisms underlying RI- and RCI-induced brain injury in mice may have some disparities that have yet to be fully unfolded.

## Data Availability

The data presented in this study are available from the authors on reasonable request.
